# Shame and anger differentially predict disidentification between collectivistic and individualistic societies

**DOI:** 10.1371/journal.pone.0289918

**Published:** 2023-09-06

**Authors:** Isabel Bierle, Julia C. Becker, Gen Nakao, Steven J. Heine

**Affiliations:** 1 Department of Psychology, University of Osnabrueck, Osnabrueck, Germany; 2 Department of Management, Otemon Gakuin University, Osaka, Japan; 3 Department of Psychology, University of British Columbia, Vancouver, Canada; Walden University, UNITED STATES

## Abstract

In the present research we tested the differential effects of anger versus shame as emotional predictors of ingroup disidentification in one rather collectivistic (Japan) and two rather individualistic societies (Germany, Canada). We tested the idea that individuals cope with socially undesired emotions by disidentifying from their group. Specifically, we predicted that after a group conflict, anger, an undesired emotion in Japan, would elicit disidentification in Japan, whereas shame, an undesired emotion in Canada and Germany, would elicit disidentification in Germany and Canada. Study 1 (N = 378) found that anger, but not shame, was related to disidentification in Japan, whereas shame, but not anger, was related to disidentification in Canada and Germany. Study 2 (N = 171) shows that, after group conflict, Japanese disidentified more when imagining to feel angry, whereas Germans disidentified more when imagining to feel ashamed. Implications for these findings are discussed.

## Introduction

We belong to a variety of social groups and derive parts of our self-evaluation from these group memberships [1–3] Nevertheless, sometimes we belong to groups that we do not wish to belong to, for example, when the group has a bad reputation [4], lacks ingroup support [5], or when there is a perceived conflict of values between oneself and the group [4, 6]. If we cannot leave this unpleasant group, one coping strategy is to psychologically distance ourselves, that is, to *disidentify* from the group [6, 7].

The aim of the present paper is to examine emotional predictors of disidentification across cultures. So far, emotional predictors of disidentification have largely been studied in a rather generalized way and exclusively within individualistic cultures. Given that cross-cultural research has shown that collectivistic and individualistic cultures differ in how emotions are valued [8], we suspect different emotions would be related to disidentification across cultures. In the present work, we focus on anger and shame. Shame has been an important variable predicting disidentification in individualistic contexts in earlier work [5], whereas anger might be a relevant emotion eliciting disidentification in more collectivistic countries as outlined below.

### Disidentifcation and emotions in individualistic versus collectivistic societies

Disidentification is the active distancing from a group that is relevant to the self-concept [4, 5, 9–12] and it consists of three components: *disengagement* (a negative motivational state that ranges from a rather passive and cognitive alienation to an active separation from one´s ingroup), *dissimilarity* (the degree to which individuals perceive themselves as different from the ingroup prototype) and *dissatisfaction* (a negative evaluation of one´s group membership; [5]). Individuals who disidentify from their groups continue to formally belong to the groups but have internally distanced themselves from them; they see themselves to be different from other group members and are dissatisfied with their group membership (e.g., [5, 12]). Disidentification is more than low identification or a lack of identification (nonidentification), because it represents an active and unique psychological state (e.g. [5, 9–11]. This means, disidentification involves disconnecting (typically negative) aspects of the group from the self, separating one’s identity from the group, feeling dissatisfied about belonging to the group and perceiving oneself as different from other group members. In contrast, nonidentification with a group is a rather neutral process which is characterized by a lack of both identification and disidentification [13]. Up until now, different antecedents of disidentification have been studied: For example, it is especially induced by negative experiences with a group, such as rejection [12], a bad reputation [4], a lack of ingroup-support [14], a perceived conflict of values between oneself and the group [6] or when an identity is under threat [15–17].

However, there have only been a few studies on *emotional predictors* of disidentification. One study conducted with German students found that negative emotions in general (an average score including anger and shame) mediated the link between un-preferred group assignment (being in a group participant did not feel they should belong to) and disidentification [18]. It remains unclear, however, whether there were any differential effects of anger and shame, because the analyses were conducted on a score that collapsed across particular kinds of emotions. Becker and Tausch illustrated that dissatisfaction was associated with shame and shame predicted intentions to conceal one´s group membership [5]. However, past research on emotional predictors of disidentification has been limited to studies of people in individualistic cultures. Recently, Bierle, Becker & Ikegami, explored whether emotional correlates of disidentification varied between individualistic and collectivistic cultures [19]. They found that disidentification exists in both individualistic and collectivistic countries but appears to serve different psychological functions: In more collectivistic cultures disidentification was used as a coping strategy to deal with intra- and intergroup conflicts. For example, when Japanese participants experienced anger because of a group conflict, they were more likely to disidentify from their ingroup. Thus, in more collectivistic societies, such as Japan, disidentification serves as a useful coping strategy to deal with culturally undesired emotions (i.e., anger) while still maintaining ingroup harmony. In contrast, in more individualistic societies, such as Germany, direct confrontation with others was used as a coping strategy to deal with intra- and intergroup conflicts. For example, when German participants experienced anger because of a group conflict, they were more likely to openly confront members of their group [19]. These findings raise the question of why these different coping strategies are used across cultures. Why would Japanese be more likely to disidentify from their ingroup when they experienced anger, when Germans tend to openly express their anger towards their ingroup?

### Anger and shame in collectivist versus individualist societies

Anger and shame are both implicated in relationships as they can be elicited by other people, by an ingroup or outgroup. Individuals feel angry when they are affronted by others, their ingroup or outgroup, and they experience shame when they fail in the eyes of others. However, cultures differ in some of the ways that relationships are formed. In more individualistic cultures, maintaining individual autonomy in relationships tends to be emphasized, meaning that people have the tendency to see themselves as independent from others, aim to pursue their individual goals actively, are motivated to feel good about themselves and focus on individuals’ needs [20–22]. In contrast, in more collectivistic cultures, achieving a sense of belongingness is emphasized [23], meaning that social norms tend to focus on group needs, interdependence, and social harmony [24]. As a consequence, it is likely that people from collectivistic and individualistic cultures experience anger and shame in interpersonal and intergroup situations somewhat differently. Indeed, much research finds that the social desirability of experiencing and expressing anger and shame varies across cultures.

For example, past research in some collectivistic cultures finds that expressing anger is less socially accepted [25, 26] and is experienced to a lesser extent than in individualistic cultures [27–29]. In fact, anger poses a serious threat to the central collectivistic goal of relational harmony and embeddedness, not at least because in collectivistic cultures, people’s close relationships are less likely to change, even those with significant conflict [30–32]. Consequently, self-assertion or expressing anger in group situations is perceived as undesirable by fellow group members as it makes it problematic for fitting in well with others. In order to comply with social norms, such as being polite, maintaining harmonious relationships, and showing emotional restraint, people in collectivistic societies avoid expressing their anger, and often rely on subtle and passive strategies instead [28, 29, 33–36]. Therefore, disidentification with one’s group may provide a way to psychologically escape the anger-eliciting group, and may function as a regulatory response to contain the disruptive emotion and limit its potentially harmful consequences [37]. We thus hypothesize that in more collectivistic societies, anger elicited by a group conflict should predict more disidentification with one’s group, because it allows to psychologically detach their selves from the ingroup so that the anger becomes less intense.

In contrast, from the perspective of individualistic values, *anger* supports goal pursuit and self-assertion and is perceived to be constructive [38–42]. In individualistic societies, experiencing and expressing anger is understood as a desirable sign of healthy and mature self-expression [37, 43–45], whereas not expressing one’s anger is perceived as potentially harmful in the long run [46]. In contrast, in collectivistic societies, individual self-expression is not as important, but instead, the relational aspect, namely managing the relationship between the self and the group is given primary importance. Supporting this argument, research has found that Chinese-Canadians experienced anger less intensely than European-Canadians and were more comfortable with strategies that served to reduce their experience of anger, whereas the European-Canadians suffered from physiological consequences if they did not openly express anger [28, 29, 47]. Thus, people in more individualistic cultures tend to be more likely to directly express their anger rather than disguising their anger by coping methods such as disidentifying with their groups. Therefore, we hypothesize that anger and disidentification should be less strongly related in individualistic cultural contexts compared with collectivistic ones.

On the other hand, *shame* is less connected to the goal of actively combating injustice through action intentions or of confronting responsible actors [38]. Instead, shame is often experienced when an individual attributes some failure or wrongdoing to internal, global, and stable aspects of the self [48], which should be particularly unpleasant in more individualistic cultures [43]. As the experience of shame highlights both the shortcomings of the individual and their dependence on other people’s judgments [49, 50], it should interfere with cultural goals of standing out and achieving high self-esteem. Hence, in individualistic cultures, shame should be expected to motivate people to hide, withdraw, or escape from the situation or public awareness [51]. Given its particular undesirability in individualistic cultures, people who experience shame need strategies to cope such as down-regulating their shame [51–53], or transforming it into a more acceptable experience [54, 55]. Hence, in more individualistic cultures, feeling ashamed because of a group conflict should lead to disidentification, because disidentification could help individuals to psychologically distance themselves from the group, thereby reducing their feelings of shame.

In contrast, in collectivistic contexts, *shame* is a more constructive and valued emotion that helps to ensure social control, and facilitates harmonious social relationships by making people aware of their shortcomings, which allows them to adjust their behaviors [50, 56, 57]. In such contexts, shame may be up-regulated and promoted, such that it is more commonly experienced. Moreover, if shame plays a role in restoring or repairing relationships in collectivistic contexts, there may be less of an urge to suppress the emotion. The typical response may instead be to yield to feelings of shame and to capitalize on their relationship-restoring potential by seeking closeness with others [58]. Hence, we expect that people in collectivistic cultures would be less likely to try to reduce their feelings of shame, nor use disidentification as coping strategy.

### The present research

We conducted 2 studies to examine whether anger and shame differentially predicted disidentification among people in more individualistic cultures (Study 1: Canada and Germany, Study 2: Germany) and in a more collectivistic culture (Study 1 and 2: Japan). Our selection of countries was guided by Hofstede’s individualism-collectivism dimension [59]. This bipolar dimension ranges from 0 to 100; higher scores indicate stronger manifestations of individualism. Germany and Canada represent individualistic countries (scores 67 and 80), whereas Japan characterizes a mid-range collectivistic country (score 47). Although Japan does not represent an extreme collectivistic country based on this dimension (and becomes more individualistic) central cultural norms are still collectivist in Japan [60]. There is a wide range of research showing the persistence of collectivism in Japan, especially the importance of collectivistic living (increased importance in social obligation, social harmony, and social contribution and decreased importance in individual rights [60–62], and low levels of relational mobility [32].

Study 1 explored the relationships between anger, shame and disidentification by exposing participants to 4 different conflict scenarios and asking them to report the degree that they would feel anger, shame, and disidentification. When selecting suitable scenarios, we were interested in situations which occur in both Japan and Germany and are thus realistic in both countries and likely to trigger authentic emotions in Japanese and German participants. In addition to that we selected scenarios which differed in group size and group type, because disidentification occurs when the group is disliked but still important to the self-concept. If one is indifferent to a negatively rated group, there should be a different emotional reaction to it. Cross cultural research has shown that the importance of groups to ones’ self is also influenced by the culture in which we grow up: specifically, people in rather individualistic cultures are by tendency intergroup oriented, that is they identify themselves with the ingroup as an abstract social category and look at the ingroup as a monolithic social category comprised of members who share similar attributes in the comparative context with outgroups. On the other hand, people in rather collectivistic cultures are by tendency intragroup oriented, that is they perceive their self to be related to other ingroup members via relational ties and the ingroup to be a bounded network of such ties [26].

Therefore, given the cultural differences regarding group and conflict types, we selected four different scenarios in Study 1 that vary in group and conflict type instead of confining us to one single scenario that might have more relevance in one specific cultural context. Study 2 was designed to replicate the finding of Study 1 by systematically testing the causal effect of anger and shame as emotional predictors of disidentification.

## Study 1

### Method

#### Participants

The samples were all undergraduate students from the University of Osnabrueck in Germany, the University of British Columbia in Canada, and Kyoto University in Japan. The study meets the strict ethical guidelines of the universities of Osnabrueck, British Columbia and Otemon Gakuin and was furthermore approved by the Office of Research Services Behavioural Research Ethics Board of the University of British Columbia. In addition to that, written informed consent was obtained from all participants. In Germany, 138 students completed the questionnaire, however, 10 participants were excluded for not having German nationality (6), having lived abroad for more than five years (3), or having more than 10% missing values (1). The final German sample included 128 participants aged 18–42 years (*M* = 22.87, *SD* = 4.39), 85.2% were female. In Japan, 133 students completed the questionnaire, however, 6 participants were excluded for not indicating their nationality (3), not having Japanese nationality (1), having lived abroad for more than five years (1) or, having more than 10% missing values (1). The final Japanese sample included 127 participants aged 18–32 years (*M* = 21.58, *SD* = 2.47), 38.96% were female. In Canada, 131 students completed the questionnaire, however 8 participants were excluded for not indicating their nationality (2), not having Canadian nationality (3) or having lived abroad for more than 5 years (3) (no Canadians had more than 10% missing values). The final Canadian sample included 123 participants aged 18–26 years (*M* = 19.89, *SD* = 1.78), 74.4% were female (2 did not indicate their sex). A sensitivity power analysis was conducted to run a regression analysis (with two predictors) for each of the individual samples using G*power [63], which indicated that each sample had 90% power to detect an effect size of at least *f*^2^ = 0.15, corresponding to a moderate effect.

#### Materials

All participants completed an online version of the questionnaire (some of the Canadians completed a printed version of the questionnaire instead). Questionnaires were administered to participants in their native language. The German version of the survey was first translated into English by the first author, checked for accuracy by an English native speaker blind to the hypotheses and then translated into Japanese by the third author and checked for accuracy by students of the third author´s team. In the whole translating process, discrepancies were resolved in discussions.

#### Scenarios

In order to show that anger vs. shame are related to disidentification in Japan vs. Canada/Germany across a diverse number of conflict scenarios, participants were presented with a scenario which represented 1 of 4 different types of group conflicts. Participants were randomly assigned to be presented with a scenario describing one of the following: a) an intragroup conflict concerning a small group (INTRA_small), b) an intergroup conflict concerning a small group (INTER_small) c) an intragroup conflict concerning a large group (INTRA_large), or d) an intergroup conflict concerning a large group (INTER_large). The material was adapted on prior work published in Bierle et al. [19], and were developed to be applicable and relevant in their respective cultural contexts. In particular, we were interested in situations that are realistic in all three countries and are likely to trigger authentic emotions among Japanese and German / Canadian participants. Since studies have shown that conflicts regarding small and large groups or intergroup and intragroup related conflicts are potentially differentially threatening to people in collectivistic and individualistic cultures [1, 64, 65], we wanted to develop scenarios that contained all four possible combinations in order to be able to generalize the results. We have the same predictions for all four different scenarios. In the INTRA_small scenario participants read that their friends were speaking badly about him/herself in a moment when they thought they were unheard. In the INTER_small scenario, participants read that an unknown group of friends were speaking badly about the participant´s own group of friends. The INTER_large scenario contained information indicating that the participant’s university fared worse than a relevant other university regarding a graduates’ chances on the job market and the possibility to get prestigious internships. In the INTRA_large scenario participants learned that students from their own university had behaved inappropriately and disrespectfully during an evening symposium with many top-ranking visiting lecturers and students of other universities (the full text of the scenarios is provided in S1 Table in S1 File. Before participants read one of four scenarios, we sampled identification with the group—we do not report these due to low reliabilities).

*Emotions*. After each scenario, participants reported how *angry* and how *ashamed* they would be in the given scenario situation. The particular items were rated on a 6-point rating scale ranging from 1 (*not at all*) to 6 (*very much)*.

*Disidentification*. Disidentification was assessed by adapting 9 items following Becker and Tausch [5] (e.g. “I feel a distance between myself and my friends/and other students of [University´s name]”; “I regret that I belong to this group of friends/to the [University´s name] student body”; “I’m completely different from most of my friends/ from most other “[University´s name] students”. Items were rated on a 6-point rating scale ranging from 1 (I totally disagree) to 6 (I fully agree) and were reliable across countries (Germany: α = .82-.92, Japan: α = .81-.87; Canada: α = .73-.91). At the end of the questionnaire, participants completed some demographic questions.

### Results and discussion

Measurement invariance was not established. Given the lack of invariance, we used a careful approach, analyzed data within countries only and avoided comparisons between countries (Results can be obtained from the first author).

We tested predictors of disidentification using anger and shame as predictors in regression models for each country and conflict situation separately. An initial analysis revealed that results were not moderated by gender (see S2 Table in S1 File). Corresponding descriptive statistics are provided in Table 1 and intercorrelations among all study variables are provided in Table 2 (intercorrelations among all study variables for each scenario separately are provided in S3 Table in S1 File). The variance inflation factor suggests that we do not have to deal with multi-collinearity.

**Table 1:** Means and standard deviations of anger, shame and disidentification (Study 1).

	Anger	Shame	Disidentification
**Canada**			
INTRA_ S	4.65 (1.38)	4.12 (1.51)	3.86 (0.98)
INTER_S	4.03 (1.45)	2.52 (1.08)	2.49 (0.86)
INTRA_L	3.82 (1.33)	4.00 (1.21)	3.50 (0.54)
INTER_L	3.87 (1.22)	2.96 (1.33)	2.46 (0.83)
**Germany**			
INTRA_ S	4.90 (1.37)	3.74 (1.50)	3.65 (1.21)
INTER_S	4.53 (1.32)	2.28 (1.11)	1.91 (0.58)
INTRA_L	3.43 (1.59)	3.97 (1.24)	3.22 (0.98)
INTER_L	3.19 (1.42)	2.25 (1.31)	2.42 (0.79)
**Japan**			
INTRA_ S	3.56 (1.68)	4.60 (1.25)	4.22 (0.75)
INTER_S	4.16 (1.46)	2.76 (1.09)	3.00 (0.87)
INTRA_L	3.59 (1.71)	3.86 (1.46)	3.11 (0.77)
INTER_L	2.64 (1.37)	2.52 (1.28)	2.63 (0.85)

Numbers in parentheses are the standard deviations. INTRA_S = intragroup conflict concerning a small group, INTER_S = intergroup conflict concerning a small group, INTRA_L = intragroup conflict concerning a large group, INTER_L = intergroup conflict concerning a large group.

**Table 2:** Intercorrelations among all study variables (Study 1).

	Shame	Disidentification
**Canada**		
Anger	.24**	.01
Shame	1	.54**
Disidentification		1
**Germany**		
Anger	.40**	.19*
Shame	1	.63**
Disidentification		1
**Japan**		
Anger	.31**	.46**
Shame	1	.45**
Disidentification		1

*p < .05;

**p < .01;

^Ϯ^ < .10 (two tailed).

Among Japanese, anger was positively related to disidentification in all 4 conflict situations (see Table 3). In contrast, shame was not related to disidentification for the INTER_small, INTRA_large and INTER_large group scenarios. In the INTRA_small scenario, however, shame did positively predict disidentification, which was counter to our hypotheses.

**Table 3:** Main effect model for prediction of disidentification for each conflict situation (Study 1).

						Collinearity Statistics	
	b	SE	*t* (df)	*p*	95% CI	Tolerance	VIF
**Canada**							
INTRA_S							
Anger	0.11	0.13	0.86 (2)	.399	-0.16; 0.39	.991	1.009
Shame	0.25	0.12	2.09 (2)	.048	0.00; 0.51	.991	1.009
INTER_S							
Anger	-0.13	0.09	-1.31 (2)	.201	-0.33; 0.07	.996	1.004
Shame	0.46	0.13	3.59 (2)	.001	0.19; 0.73	.996	1.004
INTRA_L							
Anger	-0.09	0.08	-1.13 (2)	.266	-0.26; 0.75	.621	1.611
Shame	0.21	0.09	2.34 (2)	.026	0.03; 0.40	.621	1.611
INTER_L							
Anger	-0.28	0.11	-2.45 (2)	.020	-0.51; -0.05	.978	1.023
Shame	0.22	0.10	2.07 (2)	.047	0.00; 0.43	.978	1.023
**Germany**							
INTRA_S							
Anger	0.12	0.14	0.85 (2)	.402	-0.17; 0.41	.940	1.064
Shame	0.41	0.13	3.18 (2)	.004	0.15; 0.68	.940	1.064
INTER_S							
Anger	-0.15	0.08	-1.95 (2)	.059	-0.31; 0.00	.804	1.244
Shame	0.19	0.09	2.00 (2)	.050	0.00; 0.37	.804	1.244
INTRA_L							
Anger	0.02	0.13	0.12 (2)	.905	-0.25; 0.28	.641	1.561
Shame	0.37	0.17	2.22 (2)	.035	0.03; 0.71	.641	1.561
INTER_L							
Anger	-0.13	0.10	-1.27(2)	.215	-0.34; 0.08	.507	1.971
Shame	0.51	0.11	4.50 (2)	< .001	0.28; 0.75	.507	1.971
**Japan**							
INTRA_S							
Anger	0.30	0.06	4.67 (2)	< .001	0.16; 0.43	.992	1.008
Shame	0.18	0.08	2.11 (2)	.047	0.00; 0.36	.992	1.008
INTER_S							
Anger	0.24	0.11	2.08 (2)	.049	0.00; 0.47	.867	1.153
Shame	0.22	0.15	1.46 (2)	.158	-0.09; 0.54	.867	1.153
INTRA_L							
Anger	0.19	0.06	3.08 (2)	.004	0.07; 0.32	.800	1.250
Shame	0.11	0.07	1.49 (2)	.143	-0.04; 0.26	.800	1.250
INTER_L							
Anger	0.24	0.11	2.12 (2)	.036	0.02; 0.47	.943	1.060
Shame	-0.12	0.12	-1.03(2)	.313	-0.36; 0.12	.943	1.060

INTRA_S = intragroup conflict concerning a small group, INTER_S = intergroup conflict concerning a small group, INTRA_L = intragroup conflict concerning a large group, INTER_L = intergroup conflict concerning a large group, VIF = variance inflation factor.

Among Germans, shame positively predicted disidentification in the INTRA_small, INTER_large and INTRA_large scenarios, and was in the same direction for the INTER_small scenario, although this effect only approached significance. In contrast, anger did not predict disidentification in any of the scenarios, and showed a marginal negative relation with disidentification in the INTER_small scenario.

Among Canadians, shame positively predicted disidentification across all 4 scenarios. In contrast, anger did not positively predict disidentification in any of the scenarios, and was negatively related to disidentification in the INTER_large scenario.

In sum, for the most part, the results were consistent with our hypotheses. Given that we found comparable effects across the four heterogenous scenarios, we are confident that the results can be generalized to different situations involving small and large groups, intra- and intergroup conflicts. While anger predicted disidentification among Japanese participants for all scenarios, shame was unrelated to disidentification across scenarios with the exception of the INTRA_small scenario. In stark contrast, among both the German and Canadian samples, anger did not positively predict disidentification for any of the scenarios, however, shame positively predicted disidentification for all scenarios. These findings provide preliminary support for our hypotheses and suggest that anger but not shame is related to psychological ingroup detachment for individuals in collectivistic cultures, whereas shame but not anger is related to psychological ingroup detachment for individuals in individualistic cultures.

One limitation of these findings is that we measured anger, shame and disidentification and can only speculate in terms of the causal directions. While we have reasoned that it makes sense to expect that people’s emotional reactions will lead them to disidentify it is also possible that it is the experience of disidentification that triggers anger in more collectivistic and shame in more individualistic cultures. Study 2 was conducted to address this limitation by testing the causal impact of emotions on disidentification in an experiment.

## Study 2

The aim of Study 2 was to replicate the findings of Study 1 by systematically investigating differential causal effects of anger and shame on disidentification across Japanese and German samples. Since we found a similar pattern regarding emotional predictors of disidentification among all scenarios used in Study 1, in Study 2 we concentrated on one scenario. We exposed participants to an ingroup-related conflict, induced shame or anger, and asked them how much they would disidentify from their ingroup. We expected that Japanese who were asked to focus on anger would be more likely to show disidentification compared to those who were asked to focus on shame. In contrast, we expected that Germans who were asked to focus on shame would be more likely to show disidentification compared to those who were asked to focus on anger.

### Methods

#### Participants

As in Study 1, all participants were students in metropolitan areas, to achieve comparable samples. The study meets the strict ethical guidelines of the universities of Osnabrueck, British Columbia and Otemon Gakuin. In addition to that, written informed consent was obtained from all participants. Again, they were recruited in undergraduate psychology classes and asked to report on “one´s own feelings and reactions towards other people’s behavior”. A power analysis conducted for 2x2 ANOVA using the G*power program revealed that we need 171 participants to detect an effect size of *f = 0*.*25*, which corresponds to a moderate effect. In Germany, 110 undergraduate students from the University of Osnabrueck completed the questionnaire. We excluded 10 participants because they did not have German nationality (2), said, that they did not read the situation carefully (3), had more than 10% missing values (1) or failed the manipulation check, meaning that, they did not respond to our emotion induction (e.g., when they wrote they would not be angry in the anger condition, but felt ashamed) (4). The final German sample included 100 participants aged 18–35 years (*M* = 22.61, *SD* = 3.66), 89.9% were female. In Japan, 83 undergraduate students from the Otemon Gakuin University (Osaka) completed the questionnaire. We excluded 12 participants from the analysis because they did not indicate their nationality (6), did not have Japanese nationality (4), or had more than 10% missing values (2), (no Japanese failed to the manipulation check). The final Japanese sample included 71 participants aged 18–21 years (*M* = 18.67, *SD* = 0.86), 45.1% were female. A sensitivity power analysis was conducted to run independent *t* tests for each of the individual samples using G*power [63], which indicated that each sample had 90% power to detect an effect size of at least *d* = 0.70 corresponding to a large effect.

#### Measures

Questionnaires were administered online to participants in their native language. The German version of the survey was first translated into English and then into Japanese. A team of bilingual researchers carefully checked the adequacy of translation and discrepancies were resolved in discussions.

#### Scenarios

In order to keep the questionnaire short, we included one instead of four scenarios and selected a scenario that directly related to student’s lifes. All co-authors agreed that the scenario was applicable and relevant in their respective cultural context.

The scenario containing a conflict situation was as follows: “Imagine you and a few other close classmates from your semester have been awarded a scholarship to study a semester at another university in another country. Although you know, Japanese [German] students tend to be less welcome in this country, you are looking forward to having been given this opportunity together. You are having a good time together abroad and you have even managed to find contact with local fellow students, despite the unfavorable image that prevails about students from Japan [Germany]. When it comes to the exam period, you arrange to meet with the local classmates to study together in the library. On one of the studying sessions, you sit with some of the local classmates at a common table in the library. At the next table, hidden behind a row of bookshelves, you can hear a group of students obviously behaving badly: from their corner you can hear loud conversations, cell phone beeps, and clinking drinking bottles. Once you glance through a gap in the bookshelf, you suddenly realize that the misbehaving student group consists of your Japanese [German] classmates.” After reading the scenario, participants were asked whether they had read the scenario carefully.

*Manipulation of shame and anger*. We induced feelings of shame or anger by asking participants to imagine that they felt much shame or anger (shame condition: “Imagine that a huge wave of shame arises in you and gives you a feeling of deep self-consciousness. Imagine how ashamed you feel about how your close classmates behaved in the library”, anger condition: “Imagine that a huge wave of anger arises in you. Imagine how angry you feel about how your close classmates behaved in the library”). Participants were asked to take some time to immerse themselves in the respective emotion. In order to strengthen this manipulation and to control whether participants experienced the feelings, participants were asked to describe their feelings of either being ashamed or being angry. As a manipulation check, we then checked the answers of the participants whether they described their emotion corresponding to the emotion condition (anger vs. shame) they were assigned to. In case they did not respond to our emotion induction (e.g., when they wrote they would not be angry in the anger condition, but felt ashamed), participants were excluded. As described above, 4 German but no Japanese participant failed the manipulation check, meaning that the majority of the participants (100% of the participants in Japan and 96.36% participants in Germany) answered appropriately to the manipulation, so we can be sure that one half of the participants was dealing with anger and the other half with shame.

*Disidentification*. Disidentification was assessed using the same 9 item scale as in Study 1 (all αs > = .80, Germany: Anger Condition *α* = .89, Shame Condition *α* = .88; Japan: Anger Condition *α* = .94, Shame Condition *α* = .92).

At the end of the questionnaire, participants completed some demographic questions.

### Results and discussion

Measurement invariance was not established. Given the lack of invariance, we made again only within country comparisons. As before, results were not moderated by gender (see S4a and S4b Table in S1 File).

Then, an independent-samples t-test was conducted separately for each country (instead of a 2x2 ANOVA) to compare the effect of condition (anger vs. shame) on disidentification (see Table 4 for descriptive statistics). There was a significant effect of condition on disidentification in both countries (Germany: *t*(98) = -2.91, *p* = .004, *d = 0*.*58*; Japan: *t*(69) = 2.57, *p* = .012, *d* = 0.61). In Germany, participants assigned to the shame condition reported higher levels of disidentification than participants assigned to the anger condition. In contrast in Japan, participants assigned to the shame condition reported lower levels of disidentification than respondents assigned to the anger condition. In sum, Japanese disidentified more when they felt anger compared to shame, whereas Germans disidentified more when they felt shame compared to anger.

**Table 4:** Means (*M*) and Standard Deviations (*SD*) of disidentification (Study 2).

	Germany			Japan		
	*M*	*SD*	*n*	*M*	*SD*	*n*
**Anger Condition**	2.45	0.87	49	2.98	1.16	33
**Shame Condition**	2.99	0.96	51	2.34	0.94	38

## General discussion

The aim of our research was to examine the emotional predictors of disidentification in individualistic and collectivistic cultures. Prior research has been exclusively conducted in individualistic cultures, and has studied emotional predictors of disidentification in a rather generalized way. However, due to cultural differences in how emotions are valued [8] we assumed different emotions would be related to disidentification across cultures: People try to avoid emotions that are detrimental to their central goals, which are shaped by values within their particular cultural contexts, and they seek out emotions that promote these goals [37]. We reasoned that anger should be a relatively devalued emotion in more collectivistic cultures as it stands to threaten group harmony, whereas shame should be more valued as it could facilitate the maintenance of group harmony [8]. On the other hand, we reasoned that shame should be problematic in individualistic cultures and thus people would be motived to avoid it, whereas anger could serve to help achieve personal goals [8]. The results of both studies provide evidence consistent with our hypotheses. Study 1 found that in Japan anger, but not shame, was related to disidentification. In contrast in Germany and Canada it was shame, not anger, that predicted disidentification. Likewise, Study 2 further demonstrated that Japanese disidentified more when they imagined feeling angry than when they imagined feeling ashamed, whereas Germans showed more disidentification when they imagined feeling ashamed compared with feeling angry.

The present results are in line with research on “humiliated fury,” which corresponds to shame related anger: Kirchner and colleagues found that Americans transformed high-intensity shame into anger, because shame was perceived as intolerably painful [8]. In contrast, for Japanese participants, there was no need to avoid the experience of shame (or replace it with culturally devalued anger), because shame was perceived as a valued emotion that communicates an awareness of one’s own shortcomings and helps to maintain social harmony. Although we would suggest that shame was not transformed into anger among Japanese participants in our studies, of course, we cannot completely rule out that shame was transformed into any other emotion among Japanese participants.

There are several theoretical and practical implications of the present work. First, our work informs research and theory on disidentification and broadens the perspective to a cultural context outside individualistic countries. So far, we knew that shame is related to disidentification [5] and that anger is not related to disidentification in individualistic contexts but that anger leads to confrontation intentions [19]. The current work shows that these findings cannot be generalized to other cultures, but are confined to rather individualistic countries. The major contribution of the present work is the finding that the emotional antecedents of disidentification are contrary in the examined individualistic vs. collectivistic contexts. In Japan, it was anger but not shame, whereas in Canada and Germany it was shame, but not anger, that fostered disidentification. Thus, the present work provides a better understanding of disidentification as a coping strategy to deal with culturally undesired emotions.

Secondly, the present work has culturally-specific implications for Social Identity Theory [26]. Coming back to the three subcomponents of disidentification [5]: disengagement (that ranges from a cognitive alienation to an active separation from one´s ingroup), dissimilarity (the degree to which individuals perceive themselves as different from the ingroup prototype) and dissatisfaction (a negative evaluation of one´s group membership) illustrates that high levels on each subcomponent can have quite negative implications for the group. If groups should work well, our results suggest that in order to avoid reduced levels of identification, group leaders in individualistic contexts should avoid that people feel ashamed because of their group, whereas group leaders in collectivistic contexts should avoid that people becoming angry because of their group membership.

Third, although group identification has many positive aspects for individuals and their groups [66–81], and we also provided recommendations how disidentification could be avoided, it is important to acknowledge that disidentification from certain groups can be positive and can address societal problems. For instance, prior work [68] illustrated that liberal-leaning White Americans showed disidentification from their White ingroup because they wanted to cut the association with Donald Trump and his anti-egalitarianism. In turn, racial disidentification predicted greater signaling of egalitarian beliefs and behaviors, for instance donations to racial equity-focused organizations. Thus, disidentification from anti-egalitarian groups and their proponents can lead to increased willingness to engage for more social justice. Following this, from a social justice perspective, it can have positive consequences if Japanese experience anger (instead of shame) and Germans and Canadians experience shame (instead of anger) because of an anti-egalitarian group: this feeling should lead to stronger levels of disidentification and more support for pro-egalitarian actions.

### Limitations and future directions

We acknowledge several limitations of our work. First, we did not measure whether people have an independent or interdependent self-construal but used their cultures as a proxy for the degree of individualism/collectivism. However, self-construals can differ within cultures [23]. Therefore, future research should include scales assessing self-construal, which can be used as potential mediators. Second, we focused on individualism versus collectivism in our studies for theoretical reasons, but there are others cultural dimensions that might be relevant such as power distance, uncertainty/avoidance, masculinity/femininity, long-term orientation, and indulgence/restraint [59], in explaining the differences between Japan and Germany. Future research could attempt to replicate the present findings by comparing countries that differ on collectivism and individualism but not on other cultural dimensions.

Second, we selected Japan as collectivistic country. However, recent research illustrates that although central cultural norms are still collectivist in Japan, Japan is getting more individualistic [60], but particularly in the North of Japan [40], where we did not collect our data. Nevertheless, future research could examine emotional predictors of disidentification in a society scoring very low on the individualism scale (e.g., Ghana, Burkina Faso or Indonesia). Furthermore, it would be interesting to involve participants from a collectivistic honor culture—in addition to a collectivistic face culture (like Japan). In contrast to collectivistic face cultures, in unstable and dynamic hierarchies that characterize honor cultures, achieving and protecting honor may require competitive actions to assert and protect one’s own reputation, the reputation of families, friends, or other close social groups [69, 70]. This means, if there is a situation that provides a threat to lose reputation, people in honor cultures can be expected to act competitively or even aggressively [71–73]. This would mean that anger might be more accepted in honor cultures, which could lead to similar results as in individualistic cultures.

Third, in Study 1 we measured emotions using only one item per study. We did this in order to avoid ambiguity. We are not aware of synonym of shame in German language. Moreover, this procedure is in line with several other studies [74–76]. However, future research could try to capture emotions related to anger and shame using additional descriptive items.

Fourth, we have neglected the potential role of social status. Some past research indicates that Japanese people of higher social status are more likely accorded the privilege of expressing anger to maintain hierarchies [77]. Thus, the present findings could be moderated by social status and might be particularly true for people with lower social status in Japan. Therefore, future research would benefit from testing social status as a potential moderator.

Fifth, given that we had specific predictions regarding the emotional predictors in Japan vs. Canada/Germany in Study 1, and focused on the difference between Japan vs. Germany in Study 2, we did not include a neutral baseline group. Future studies that included neutral baseline groups may be informative. Future research should also examine intra-individual differences, that is, to assess identification and disidentification at Time 1, then elicit one emotion (anger vs. shame) and test whether there are intra-individual changes in disidentification and identification based on the emotion that was elicited.

Finally, it is possible, that the emotion participants were asked to imagine did not fit their natural emotional appraisal of the scenario situation. Therefore, it would be helpful if future research could extend the manipulation check by asking participants how strongly they felt the emotion that we induced (and potential other emotions). Furthermore, building on our findings, future studies could use a different emotion manipulation.

### Conclusion

In sum, this research extends the literature on emotional predictors of disidentification in a number of ways. First, it is novel to examine anger and shame as specific emotional predictors of disidentification across cultures. Second, research on disidentification has thus far been largely conducted in individualistic cultures (for exceptions see [78–82]), and research has shown a fairly reliable relationship between shame and disidentification [5]. Our research demonstrates that this relationship between shame and disidentification does not generalize to all people, as in collectivistic contexts disidentification is predicted by anger. Moreover, our findings provided similar results across different conflict scenarios (i.e., inter vs. intra group and small vs. large group related conflict scenarios), and across both correlational and experimental designs, suggesting a rather robust tendency. It would be informative in future research to see if these results generalize to other individualistic and collectivistic cultures, and whether similar findings emerge with other related emotion terms.

## Supporting information

S1 File (DOCX)

## References

[1] Brewer MB, Gardner W. Who is this "We"? Levels of collective identity and self representations. J Pers Soc Psychol. 1996;71:83–93. doi: 10.1037/0022-3514.71.1.83

[2] Ellemers N. The group self. Science. 2012;336:848–52. doi: 10.1126/science.1220987 22605760

[3] Sedikides C, Brewer MB, editors. Individual Self, Relational Self, Collective Self. 0th ed.: Psychology Press; 2001.

[4] Elsbach KD, Bhattacharya CB. Defining Who You Are By What You’re Not: Organizational Disidentification and The National Rifle Association. Organization Science. 2001;12:393–413. doi: 10.1287/orsc.12.4.393.10638

[5] Becker JC, Tausch N. When Group Memberships are Negative: The Concept, Measurement, and Behavioral Implications of Psychological Disidentification. Self and Identity. 2014;13:294–321. doi: 10.1080/15298868.2013.819991

[6] Glasford DE, Pratto F, Dovidio JF. Intragroup dissonance: Responses to ingroup violation of personal values. Journal of Experimental Social Psychology. 2008;44:1057–64. doi: 10.1016/j.jesp.2007.10.004

[7] Dukerich, J. M., Kramer, R. M. & Parks, J. M. The dark side of organizational identification; 1998.

[8] Kirchner A, Boiger M, Uchida Y, Norasakkunkit V, Verduyn P, Mesquita B. Humiliated fury is not universal: the co-occurrence of anger and shame in the United States and Japan. Cognition & Emotion. 2018;32:1317–28. doi: 10.1080/02699931.2017.1414686 29283312

[9] de Vreeze J, Matschke C. Keeping up appearances: Strategic information exchange by disidentified group members. PLOS ONE. 2017;12:e0175155. doi: 10.1371/journal.pone.0175155 28384322 PMC5383236

[10] de Vreeze J, Matschke C, Cress U. Neither fish nor fowl: A perceived mismatch in norms and values between oneself, other students, and people back home undermines adaptation to university. Br J Soc Psychol. 2018;57:684–702. doi: 10.1111/bjso.12253 29527707

[11] Kreiner GE, Ashforth BE. Evidence toward an expanded model of organizational identification. J. Organiz. Behav. 2004;25:1–27. doi: 10.1002/job.234

[12] Matschke C, Sassenberg K. Does rejection lead to disidentification? The role of internal motivation and avoidance strategies. Eur. J. Soc. Psychol. 2010;40:891–900. doi: 10.1002/ejsp.756

[13] Elsbach KD. An expanded model of organizational identification; 1999.

[14] Becker JC, Tausch N, Spears R, Christ O. Committed dis(s)idents: participation in radical collective action fosters disidentification with the broader in-group but enhances political identification. Pers Soc Psychol Bull. 2011;37:1104–16. doi: 10.1177/0146167211407076 21543649

[15] Hodson G, Esses VM. Distancing oneself from negative attributes and the personal/group discrimination discrepancy. Journal of Experimental Social Psychology. 2002;38:500–7. doi: 10.1016/S0022-1031(02)00012-4

[16] Peetz J, Wilson AE, Strahan EJ. So Far Away: The Role of Subjective Temporal Distance to Future Goals in Motivation and Behavior. Social Cognition. 2009;27:475–95. doi: 10.1521/soco.2009.27.4.475

[17] Bizman A, Yinon Y. Engaging in distancing tactics among sport fans: effects on self-esteem and emotional responses. J Soc Psychol. 2002;142:381–92. doi: 10.1080/00224540209603906 12058976

[18] de Vreeze J, Matschke C. Don’t Put Me in This Group. Social Psychology. 2019;50:80–93. doi: 10.1027/1864-9335/a000363

[19] Bierle I, Becker JC, IKEGAMI T. Coping with unpleasant group memberships in Japan and Germany: Cultural differences in disidentification, confrontation and emotion regulation. Eur. J. Soc. Psychol. 2019;49:953–69. doi: 10.1002/ejsp.2562

[20] Hochschild AR. The Culture of Politics: Traditional, Postmodern, Cold-modern, and Warm-modern Ideals of Care. Soc Polit. 1995;2:331–46. doi: 10.1093/sp/2.3.331

[21] Kim H, Markus HR. Deviance or uniqueness, harmony or conformity? A cultural analysis. J Pers Soc Psychol. 1999;77:785–800. doi: 10.1037/0022-3514.77.4.785

[22] Miller PJ, Wang S, Sandel T, Cho GE. Self-Esteem as Folk Theory: A Comparison of European American and Taiwanese Mothers’ Beliefs. Parenting. 2002;2:209–39. doi: 10.1207/S15327922PAR0203_02

[23] Markus HR, Kitayama S. Culture and the self: Implications for cognition, emotion, and motivation. Psychological Review. 1991;98:224–53. doi: 10.1037/0033-295X.98.2.224

[24] Tsai JL. Ideal Affect: Cultural Causes and Behavioral Consequences. Perspect Psychol Sci. 2007;2:242–59. doi: 10.1111/j.1745-6916.2007.00043.x 26151968

[25] Ohbuchi K-I. Conflict management in Japan. New York: Wiley & Sons; 1998.

[26] Yuki M. Intergroup Comparison versus Intragroup Relationships: A Cross-Cultural Examination of Social Identity Theory in North American and East Asian Cultural Contexts. Social Psychology Quarterly. 2003;66:166. doi: 10.2307/1519846

[27] Kitayama S, Mesquita B, Karasawa M. Cultural affordances and emotional experience: socially engaging and disengaging emotions in Japan and the United States. J Pers Soc Psychol. 2006;91:890–903. doi: 10.1037/0022-3514.91.5.890 17059308

[28] Mauss IB, Butler EA. Cultural context moderates the relationship between emotion control values and cardiovascular challenge versus threat responses. Biol Psychol. 2010;84:521–30. doi: 10.1016/j.biopsycho.2009.09.010 19786064 PMC2950892

[29] Mauss IB, Butler EA, Roberts NA, Chu A. Emotion Control Values and Responding to an Anger Provocation inAsian-American and European-American Individuals. Cognition & Emotion. 2010;24:1026–43. doi: 10.1080/02699930903122273 21116444 PMC2992431

[30] Adams G. The cultural grounding of personal relationship: enemyship in North American and West African worlds. J Pers Soc Psychol. 2005;88:948–68. doi: 10.1037/0022-3514.88.6.948 15982115

[31] Li LMW, Adams G, Kurtiş T, Hamamura T. Beware of friends: The cultural psychology of relational mobility and cautious intimacy. Asian Journal of Social Psychology. 2015;18:124–33. doi: 10.1111/ajsp.12091

[32] Thomson R, Yuki M, Talhelm T, Schug J, Kito M, Ayanian AH, et al. Relational mobility predicts social behaviors in 39 countries and is tied to historical farming and threat. Proc Natl Acad Sci U S A. 2018;115:7521–6. doi: 10.1073/pnas.1713191115 29959208 PMC6055178

[33] Lee EA, Soto JA, Swim JK, Bernstein MJ. Bitter reproach or sweet revenge: cultural differences in response to racism. Pers Soc Psychol Bull. 2012;38:920–32. doi: 10.1177/0146167212440292 22496162

[34] Fischer FB, Becker JC, Kito M, Zamantılı Nayır D. Collective action against sexism in Germany, Turkey, and Japan: The influence of self-construal and face concerns. Group Processes & Intergroup Relations. 2017;20:409–23. doi: 10.1177/1368430216683533

[35] Ohbuchi K-I, Atsumi E. Avoidance Brings Japanese Employees What They Care About in Conflict Management: Its Functionality and “Good Member” Image. Negotiation and Conflict Management Research. 2010;3:117–29. doi: 10.1111/j.1750-4716.2010.00052.x

[36] Ohbuchi K-I, Takahashi Y. Cultural Styles of Conflict Management in Japanese and Americans: Passivity, Covertness, and Effectiveness of Strategies1. J Appl Social Pyschol. 1994;24:1345–66. doi: 10.1111/j.1559-1816.1994.tb01553.x

[37] Mesquita B. Emotions as dynamic cultural phenomena. New York: Oxford University Press; 2003.

[38] Averill JR. Studies on anger and aggression: Implications for theories of emotion. American Psychologist. 1983;38:1145–60. doi: 10.1037/0003-066X.38.11.1145 6650969

[39] Ellsworth P&S, K.R. Appraisal processes in emotion; 2003.

[40] Kitayama S, Ishii K, Imada T, Takemura K, Ramaswamy J. Voluntary settlement and the spirit of independence: evidence from Japan’s "Northern frontier". J Pers Soc Psychol. 2006;91:369–84. doi: 10.1037/0022-3514.91.3.369 16938025

[41] Fischer AH, Roseman IJ. Beat them or ban them: the characteristics and social functions of anger and contempt. J Pers Soc Psychol. 2007;93:103–15. doi: 10.1037/0022-3514.93.1.103 17605592

[42] Tsai JL, Miao FF, Seppala E, Fung HH, Yeung DY. Influence and adjustment goals: sources of cultural differences in ideal affect. J Pers Soc Psychol. 2007;92:1102–17. doi: 10.1037/0022-3514.92.6.1102 17547491

[43] Boiger M, de Deyne S, Mesquita B. Emotions in "the world": cultural practices, products, and meanings of anger and shame in two individualist cultures. Front Psychol. 2013;4:867. doi: 10.3389/fpsyg.2013.00867 24367340 PMC3852096

[44] Tamir M, Schwartz SH, Cieciuch J, Riediger M, Torres C, Scollon C, et al. Desired emotions across cultures: A value-based account. J Pers Soc Psychol. 2016;111:67–82. doi: 10.1037/pspp0000072 26524003

[45] de Leersnyder J, Koval P, Kuppens P, Mesquita B. Emotions and concerns: Situational evidence for their systematic co-occurrence. Emotion. 2018;18:597–614. doi: 10.1037/emo0000314 28604034

[46] Shweder R. A., Haidt J., Horton R., & Joseph C. The cultural psychology of the emotions: Ancient and renewed. Lewis W: M, Haviland-Jones J., Barret LF (red.), Handbook of emotions. New York, NY, US: The Guilford Press; 2008.

[47] Anderson, J. C., Linden, W. The influence of culture on cardiovascular response to anger: Citation poster session presented at the annual meeting of the American Psychosomatic Society. Denver, CO; 2006.

[48] Tracy JL, Robins RW. Appraisal antecedents of shame and guilt: support for a theoretical model. Pers Soc Psychol Bull. 2006;32:1339–51. doi: 10.1177/0146167206290212 16963605

[49] Kitayama S, Markus HR, Matsumoto H, Norasakkunkit V. Individual and collective processes in the construction of the self: Self-enhancement in the United States and self-criticism in Japan. Journal of Personality and Social Psychology. 1997;72:1245–67. doi: 10.1037//0022-3514.72.6.1245 9177018

[50] Lindsay-Hartz J. Contrasting Experiences of Shame and Guilt. American Behavioral Scientist. 1984;27:689–704. doi: 10.1177/000276484027006003

[51] Tangney J. P. & Fischer K. W. Self-conscious emotions. New York: Guilford; 1995.

[52] Scheff TJ. Shame and the Social Bond: A Sociological Theory. Sociological Theory. 2000;18:84–99. doi: 10.1111/0735-2751.00089

[53] Schmader T., & Lickel B. Stigma and shame: Emotional responses to the stereotypic actions of one’sethnic ingroup: Mahwah, NJ: Lawrence Erlbaum.

[54] Allyn D. I Can’t Believe I Just Did that: How (seemingly) Small Embarrassments Can Wreak Havoc in Your Life—and what You Can Do to Put a Stop to Them. New York; 2004.

[55] Bear GG, Uribe-Zarain X, Manning MA, Shiomi K. Shame, guilt, blaming, and anger: Differences between children in Japan and the US. Motiv Emot. 2009;33:229–38. doi: 10.1007/s11031-009-9130-8

[56] Ha FI. Shame in Asian and Western Cultures. American Behavioral Scientist. 1995;38:1114–31. doi: 10.1177/0002764295038008007

[57] Sznycer D., Takemura K., Delton A. W., Sato K., Robertson T., Cosmides L., et al. Cross-cultural differences and similarities in proneness to shame: An adaptationist and ecological approach. Evolutionary Psychology. 2012. 22947644 10.1177/147470491201000213 PMC3604996

[58] Mesquita, B., & Karasawa, M. Self-conscious emotions as dynamic cultural processes; 2004.

[59] Hofstede G. The dimension scores in the Hofstede model of national culture can be downloaded here. 17.08.2016. https://geerthofstede.com/research-and-vsm/dimension-data-matrix/. Accessed 31 May 2021.

[60] Ogihara Y. Temporal Changes in Individualism and Their Ramification in Japan: Rising Individualism and Conflicts with Persisting Collectivism. Front Psychol. 2017;8:695. doi: 10.3389/fpsyg.2017.00695 28588512 PMC5440576

[61] Hamamura T. Are cultures becoming individualistic? A cross-temporal comparison of individualism-collectivism in the United States and Japan. Pers Soc Psychol Rev. 2012;16:3–24. doi: 10.1177/1088868311411587 21700795

[62] Kito MI, Yuki M, Thomson R. Relational mobility and close relationships: A socioecological approach to explain cross-cultural differences. Pers Relationship. 2017;24:114–30. doi: 10.1111/pere.12174

[63] Faul F, Erdfelder E, Lang A-G, Buchner A. G*Power 3: a flexible statistical power analysis program for the social, behavioral, and biomedical sciences. Behavior Research Methods. 2007;39:175–91. doi: 10.3758/bf03193146 17695343

[64] Prentice DA, Miller DT, Lightdale JR. Asymmetries in Attachments to Groups and to their Members: Distinguishing between Common-Identity and Common-Bond Groups. Pers Soc Psychol Bull. 1994;20:484–93. doi: 10.1177/0146167294205005

[65] Vignoles VL, Chryssochoou X, Breakwell GM. The Distinctiveness Principle: Identity, Meaning, and the Bounds of Cultural Relativity. Pers Soc Psychol Rev. 2000;4:337–54. doi: 10.1207/S15327957PSPR0404_4

[66] Tajfel H., Turner J. An integrative theory of intergroup conflict. In: Austin WG, Worchel S (eds.). The social psychology of intergroup relations. Monterey, CA: Brooks/Cole; 1979. p. 33–47.

[67] Tajfel H., Turner JC. The social identity theory of intergroup conflict. In Austin W., Worchel G, S (Eds.), The social psychology of intergroup relations. Chicago: Nelson Hall,1986.p.1–17.

[68] Dai J. D., Eason A. E., Brady L. M., & Fryberg S. A. #NotAllWhites: Liberal-Leaning White Americans Racially Disidentify and Increase Support for Racial Equity. Personality and Social Psychology Bulletin, 2021; 47: 1612–632. doi: 10.1177/0146167220987988 33605186

[69] Leung AK-Y, Cohen D. Within- and between-culture variation: individual differences and the cultural logics of honor, face, and dignity cultures. J Pers Soc Psychol. 2011;100:507–26. doi: 10.1037/a0022151 21244179

[70] Rodriguez Mosquera PM, Fischer AH, Manstead ASR, Zaalberg R. Attack, disapproval, or withdrawal? The role of honour in anger and shame responses to being insulted. Cognition & Emotion. 2008;22:1471–98. doi: 10.1080/02699930701822272

[71] Cohen D, Nisbett RE, Bowdle BF, Schwarz N. Insult, aggression, and the southern culture of honor: An "experimental ethnography". J Pers Soc Psychol. 1996;70:945–60. doi: 10.1037//0022-3514.70.5.945 8656339

[72] Ijzerman H, van Dijk WW, Gallucci M. A bumpy train ride: a field experiment on insult, honor, and emotional reactions. Emotion. 2007;7:869–75. doi: 10.1037/1528-3542.7.4.869 18039055

[73] Nisbett, R. E., & Cohen, D. Culture of honor: The psychology of violence in the South: CO: Westview Press.

[74] Landmann H, Gaschler R, Rohmann A. What is threatening about refugees? Identifying different types of threat and their association with emotional responses and attitudes towards refugee migration. Eur. J. Soc. Psychol. 2019;49:1401–20. doi: 10.1002/ejsp.2593

[75] Berndsen M, Gausel N. When majority members exclude ethnic minorities: The impact of shame on the desire to object to immoral acts. Eur. J. Soc. Psychol. 2015;45:728–41. doi: 10.1002/ejsp.2127

[76] Babcock MK, Sabini J. On differentiating embarrassment from shame. Eur. J. Soc. Psychol. 1990;20:151–69. doi: 10.1002/ejsp.2420200206

[77] Park J., Kitayama S., Markus HR, Coe CL, Miyamoto Y, Karasawa M., et al. Social status and anger expression: the cultural moderation hypothesis. Emotion. 2013; 13(6):1122–1137. doi: 10.1037/a0034273 24098926 PMC3859704

[78] Chen Y-R, Brockner J, Katz T. Toward an explanation of cultural differences in in-group favoritism: The role of individual versus collective primacy. J Pers Soc Psychol. 1998;75:1490–502. doi: 10.1037/0022-3514.75.6.1490

[79] Ikegami T. Consequences of disaffected social identity: Evaluation of in-group and out-group members in the face of threats to group status. Seoul, Republic of Korea; 2004.

[80] Ikegami T. Precursors and Consequences of In-Group Disidentification: Status System Beliefs and Social Identity. Identity. 2010;10:233–53. doi: 10.1080/15283488.2010.523590

[81] Ikegami T, Ishida Y. Status hierarchy and the role of disidentification in discriminatory perception of outgroups 1. Japanese Psychological Research. 2007;49:136–47. doi: 10.1111/j.1468-5884.2007.00340.x

[82] Matschke C, Fehr J. Does Identity Incompatibility Lead to Disidentification? Internal Motivation to Be a Group Member Acts As Buffer for Sojourners from Independent Cultures, Whereas External Motivation Acts As Buffer for Sojourners from Interdependent Cultures. Front Psychol. 2017;8:335. doi: 10.3389/fpsyg.2017.00335 28326055 PMC5339250

