# Supplementary material for: Shame and anger differentially predict disidentification between collectivistic and individualistic societies
Source: PLoS One. 2023 Sep 6;18(9):e0289918. doi: 10.1371/journal.pone.0289918 (PMC10482281; doi:10.1371/journal.pone.0289918)
Supplement: S1 File — (DOCX) [file pone.0289918.s001.docx]

# Supporting information

**S1 Table. Full text of scenarios (Study 1).**

**S2 Table.** **Main effect model for Prediction of Disidentification for each conflict situation controlled for sex of the participant (Study 1).**

**S3 Table. Intercorrelations among all study variables for each scenario separately (Study 1)**

**S4a Table. Fixed-Effects ANOVA results using disidentification as the criterion controlled for sex of the participant (Study 2; Germany).**

**S4b Table. Fixed-Effects ANOVA results using disidentification as the criterion controlled for sex of the participant (Study 2; Japan).**

# Supplementary material

**Table S1. Full text of scenarios (Study 1).**

| **Scenario 1:**  **Intragroup Conflict Small Group (Friends)** | **Scenario 2:**  **Intergroup Conflict Small Group (Friends)** | **Scenario 3:**  **Intergroup Conflict Large Group**  **(University)*** | **Scenario 4:**  **Intragroup Conflict Large Group**  **(University)*** |
| --- | --- | --- | --- |
| Imagine you are sitting in the library to prepare for exams. Coming from the neighboring table, which is hidden behind a set of bookshelves, you overhear a group of students who are obviously talking negatively about another person who supposedly asks stupid questions in lectures and generally doesn´t appear to be the “brightest.“ Only by listening more closely, you recognize the familiar voices of some of your best friends. When, suddenly, your name is mentioned, you realize that those derogatory comments referred to you and that your friends are talking badly about you behind your back. | Imagine you are sitting in the library to prepare for exams. Coming from the neighboring table, which is hidden behind a set of bookshelves, you overhear a group of students who are obviously talking negatively about certain people who supposedly ask stupid questions in lectures and generally don’t appear to be the “brightest.“ Only by secretly peeking through the bookshelves, you see that the gossips are fellow students from your semester, who you are not friends with, though. When, suddenly, your friends’ names are mentioned, you realize that those derogatory comments referred to your friends and that the gossips are talking badly about them behind their backs. | Imagine you are reading in a study that *[University´s name]* students have significantly lower chances on the job market compared to *[rival University´s name]* students. *[University´s name]* students complete less prestigious internships and are generally not as qualified as *[rival University´s name]* students. *[Rival University´s name]* students receive the results of the study with joy. Critics of the study object that such results weren't surprising, since *[University´s name]* is strongly disadvantaged by the unfair distribution of money within *Canada/Germany/Japan*. On the contrary, *[rival University´s name]* is treated preferentially. | Imagine you are taking part in an evening symposium at *[University´s name]* that many top-ranking visiting guest lecturers have been invited to. The audience is broadly diverse, sitting together according to different places of origin. With the name tags also indicating one’s profession, you can see that the audience consists of students from other universities, such as *[rival University´s name]*, several *[University´s name]* students, as well as professionals. As the presenter asks for silence, coming from one corner, you can hear loud murmurs, a phone ringing and bottles falling over. When you turn around, you see the troublemakers’ name tags and realize that they are students from your university. Moreover, the group of *[University´s name]* students stood out by posing stupid questions to the lecturers. |

*The text used in the German sample referred to “students of the University of Osnabrueck” the respective rival university was “the University of Hannover”; the text used in the Japanese sample referred to “students of Kyoto University” the respective rival university was “the University of Tokyo”; the text used in the Canadian sample referred to “UBC students” the respective rival university was “UToronto”.

**S2 Table.** **Main effect model for Prediction of Disidentification for each conflict situation controlled for sex of the participant (Study 1).**

|  |  |  |  |  |
| --- | --- | --- | --- | --- |
|  | **b** | **SE** | ***t (*df)** | ***p*** |
| **Canada** |  |  |  |  |
| INTRA_S |  |  |  |  |
| Anger | 0.04 | 0.15 | 0.29 (2) | .774 |
| Shame | 0.22 | 0.13 | 1.71 (2) | .102 |
| Sex of participant | 0.45 | 0.45 | 0.99(2) | .331 |
| INTER_S |  |  |  |  |
| Anger | -0.09 | 0.12 | -0.71 (2) | .483 |
| Shame | 0.45 | 0.13 | 3.24 (2) | .004 |
| Sex of participant | -0.22 | 0.46 | -0.47 (2) | .643 |
| INTRA_L |  |  |  |  |
| Anger | -0.09 | 0.08 | -1.10 (2) | .281 |
| Shame | 0.21 | 0.09 | 2.34 (2) | .033 |
| Sex of participant | 0.13 | 0.22 | 0.58 (2) | .563 |
| INTER_L |  |  |  |  |
| Anger | -0.26 | 0.12 | -0.36 (2) | .042 |
| Shame | 0.21 | 0.11 | 1.96 (2) | .060 |
| Sex of participant | -0.18 | 0.32 | -0.57 (2) | .574 |
| **Germany** |  |  |  |  |
| INTRA_S |  |  |  |  |
| Anger | 0.20 | 0.15 | 1.36 (2) | .185 |
| Shame | 0.44 | 0.13 | 3.48 (2) | .002 |
| Sex of participant | -0.78 | 0.51 | -1.55 (2) | .131 |
| INTER_S |  |  |  |  |
| Anger | -0.13 | .08 | -1.69 (2) | .099 |
| Shame | 0.189 | 0.09 | 2.04 (2) | .050 |
| Sex of participant | -0.29 | 0.27 | -1.103 (2) | .278 |
| INTRA_L |  |  |  |  |
| Anger | -0.04 | .13 | -0.27 (2) | .784 |
| Shame | .43 | .17 | 2.53 (2) | .018 |
| Sex of participant | 0.68 | 0.49 | 1.39 (2) | .176 |
| INTER_L |  |  |  |  |
| Anger | -0.19 | 0.10 | -1.85(2) | .075 |
| Shame | 0.59 | 0.11 | 5.24 (2) | <.001 |
| Sex of participant | -0.68 | 0.31 | -2.17 (2) | .039 |
| **Japan** |  |  |  |  |
| INTRA_S |  |  |  |  |
| Anger | 0.29 | 0.06 | 4.39 (2) | <,001 |
| Shame | 0.16 | 0.08 | 1.86 (2) | .076 |
| Sex of participant | 0.12 | 0.23 | 0.56 (2) | .581 |
| INTER_S |  |  |  |  |
| Anger | 0.23 | 0.11 | 2.00 (2) | .058 |
| Shame | 0.22 | 0.15 | 1.43 (2) | .167 |
| Sex of participant | 0.13 | 0.32 | 0.39 (2) | .694 |
| INTRA_L |  |  |  |  |
| Anger | 0.19 | 0.06 | 3.04 (2) | .004 |
| Shame | 0.11 | 0.07 | 1.44 (2) | .157 |
| Sex of participant | -0.00 | 0.20 | -0.04 (2) | .965 |
| INTER_L |  |  |  |  |
| Anger | 0.2 | 0.11 | 1.98 (2) | .057 |
| Shame | -0.13 | 0.12 | -1.09 (2) | .282 |
| Sex of participant | 0.22 | 0.37 | 0.61 (2) | .544 |

**S3 Table. Intercorrelations among all study variables for each scenario separately (Study 1)**

|  | **Intra_S** | | **Inter_S** | | **Intra_L** | | **Inter_L** | |
| --- | --- | --- | --- | --- | --- | --- | --- | --- |
|  | 2 | 3 | 2 | 3 | 2 | 3 | 2 | 3 |
| **Canada** |  |  |  |  |  |  |  |  |
| Anger | .08 | .20 | .06 | -.17 | .61** | .06 | .15 | -.34* |
| Shame | 1 | .33 | 1 | .57** | 1 | .34* | 1 | .27 |
| Disidentification |  | 1 |  | 1 |  | 1 |  | 1 |
| **Germany** |  |  |  |  |  |  |  |  |
| Anger | .24 | .26 | .56* | -.17 | .67** | .41* | .70** | .36* |
| Shame | 1 | .54** | 1 | .15 | 1 | .48** | 1 | .68** |
| Disidentification |  | 1 |  | 1 |  | 1 |  | 1 |
| **Japan** |  |  |  |  |  |  |  |  |
| Anger | .09 | .68** | .36 | .49* | .44** | .53** | .23 | .35 |
| Shame | 1 | .08 | 1 | .42* | 1 | .40** | 1 | -.09 |
| Disidentification |  | 1 |  | 1 |  | 1 |  | 1 |

**S4a Table.** **Fixed-Effects ANOVA results using disidentification as the criterion controlled for sex of the participant (Study 2; Germany).**

| Predictor | Sum of Squares | df | Mean Square | *F* | *p* | Partial  Eta-Quadrat |
| --- | --- | --- | --- | --- | --- | --- |
| Intercept | 90.58 | 1 | 90.58 | 102.67 | <.001 | .519 |
| Condition | 2.59 | 1 | 2.593 | 2.93 | .090 | .030 |
| Sex | 0.01 | 2 | 0.00 | 0.00 | .994 | .000 |
| Condition * Sex | 0.00 | 1 | 0.00 | 0.00 | .979 | .000 |
| Error | 83.80 | 95 | 0.88 |  |  |  |

**S4b Table**. **S4b Table. Fixed-Effects ANOVA results using disidentification as the criterion controlled for sex of the participant (Study 2; Japan).**

| Predictor | Sum of Squares | df | Mean Square | *F* | *p* | Partial  Eta-Quadrat |
| --- | --- | --- | --- | --- | --- | --- |
| Intercept | 484.34 | 1 | 484,341 | 441.04 | <.001 | .868 |
| Condition | 6.46 | 1 | 6.46 | 5.88 | .018 | .081 |
| Sex | 0.09 | 1 | 0.09 | 0.08 | .770 | .001 |
| Condition * Sex | 2.51 | 1 | 2.51 | 2.28 | .135 | .033 |
| Error | 73.57 | 67 | 1.01 |  |  |  |
